# A Clinical Phrase Book, in English and German

**Published:** 1853-07

**Authors:** 


***A Clinical Phrase Book, in English and German.*** By Montgomery Johns, M. D.

This unpretending little book is the production of one of our young fellow townsmen, and it is a highly creditable performance. It supplies a *desideratum* which is making itself felt more and more every day. A knowledge of German and French, though absolutely essential to a medical man at the present day, is, like much more essential knowledge, possessed by very few who prefix doctor to their names. To such men, and even to those who read the German language, but who have not been accustomed to speak it, this little book recommends itself very highly. If properly studied, it will assist them out of many a disagreeable dilemma.

The phrases are numerous and contain nearly every question likely to be asked by a physician in the examination of a case of disease. Besides this, the author has subjoined a brief grammar and vocabulary, so that not a little insight into the structure of the German language can be acquired through the medium of this volume.

We must, however, caution the tyro in German against resting in any set of phrases, however full. This little book does all that can be done by a phrase-book; more than we have ever seen done before in so small a compass. But the limits which exist in the nature of things cannot be overleaped by any industry or ability. German patients, like English ones, are not always content with plain answers to simple questions, but think themselves bound to enlighten their medical advisers with their opinions upon matters and things in general, and upon their ailments in particular. However annoying such a disposition is to the man of business habits, the physician must put up with it, for he often gains more from this rambling chat, than from direct and plain answers. Like the lawyer, he is sometimes obliged to let the witness tell his own story in his own way, or run the risk of getting nothing at all out of him. Hence, as we said before, no mere phrase-book will answer the purpose of the physician. He must go on and acquire sufficient knowledge of the language to enable him to mingle in general conversation.

